# The Anomalous Influence of Spectral Resolution on Pulsed THz Time Domain Spectroscopy under Real Conditions

**DOI:** 10.3390/s17122883

**Published:** 2017-12-12

**Authors:** Vyacheslav A. Trofimov, Svetlana A. Varentsova

**Affiliations:** Faculty of Computational Mathematics and Cybernetics, Lomonosov Moscow State University, Leninskiye Gory, Moscow 119992, Russia; svarentsova@gmail.com

**Keywords:** pulsed THz time-domain spectroscopy, spectral dynamics analysis method (SDA-method), detection and identification of substances, integral correlation criteria, spectral resolution

## Abstract

We have studied the spectral resolution influence on the accuracy of the substance detection and identification at using a broadband THz pulse measured under real conditions (at a distance of more than 3 m from a THz emitter in ambient air with a relative humidity of about 50%). We show that increasing spectral resolution leads to manifestation of small-scale perturbations (random fluctuations) in the signal spectrum caused by the influence of the environment or the sample structure. Decreasing the spectral resolution allows us to exclude from consideration this small-scale modulation of the signal as well as to detect the water vapor absorption frequencies. This fact is important in practice because it allows us to increase the signal processing rate. In order to increase the detection reliability, it is advisable to decrease the spectral resolution up to values of not more than 40% of the corresponding spectral line bandwidth. The method of spectral dynamics analysis together with the integral correlation criteria is used for the substance detection and identification. Neutral substances such as chocolate and cookies are used as the samples in the physical experiment.

## 1. Introduction

Currently, THz time-domain spectroscopy (THz TDS) is a common technique for substance identification. It is widely used for security problems [1,2,3,4,5,6,7,8,9,10,11,12,13,14,15,16,17], non-destructive inspection of various materials [18,19,20], for biomedical [21,22,23,24,25] and pharmaceutical applications [26,27]. It is regarded as a powerful new tool for the chemical sciences [28,29,30] and is used for counterfeit detection [31]. Development of compact, economical, high-resolution, and high-power terahertz imaging and spectroscopy systems [32,33] are also a research focus.

At present, there are two main methods which are used for substance detection and identification. The most common method is based on the spectrum analysis of the THz signal transmitted through the substance under investigation or reflected from it. The absorption frequencies of the substance under investigation are compared with the absorption frequencies of the standard substances from database. The detection and identification occurs on the base of this comparison [1,2,3,4,5,6]. We call this method as a standard THz TDS method. We emphasize that the standard method is not a method of generation and registration of the THz signal, but rather the analysis of the spectral features of the investigated THz signal.

However, over time, it was found that this method has significant disadvantages. In particular, many hazardous substances have simulators, i.e., neutral substances with a similar set of the absorption frequencies, which makes using the standard THz-TDS method insufficiently effective. Ordinary packing materials (paper, polyethylene, cardboard, cotton, etc.), the sample’s surface inhomogeneity, high atmospheric humidity all decrease the effectiveness of this method [12,13,14,15,16,17]. Another method, proposed by us, is the spectral dynamics analysis (SDA) method [34,35], which is based on the analysis of time-dependent spectral intensity (spectral dynamics) at chosen frequencies. Its use together with various integral correlation criteria for the substance assessment in the THz signal under investigation allows detection of the dangerous substance—its presence or absence—with high probability. Nevertheless, the SDA-method also possesses certain limitations. For example, it cannot be effective if the spectral intensity evolution for two substances (dangerous and neutral) are similar. Therefore, it is very important to provide a preliminary analysis of the substance response spectrum in order to eliminate as much as possible the false absorption frequencies.

Consequently, one more important practical problem, which is studied below, is the spectral resolution influence on the accuracy of the substance detection and its identification at using the high-noise THz signal. Generally, high spectral resolution is required for a spectrometer. For THz-TDS, however, the spectral resolution is related to the measurement time (higher resolution requires longer time). When the measurement time is too long, more environmental noise is introduced into the response to the THz pulse action and thus makes the signal-to-noise ratio of the spectrometer worse. This fact essentially results in the increase of false detection.

In the current paper, we show that increasing the spectral resolution leads to the manifestation of small-scale perturbations in the signal spectrum caused by the environment influence or the sample structure. At the same time, decreasing the spectral resolution allows one to exclude from consideration the false absorption frequencies caused by small-scale modulation.

The description of the algorithm for the false absorption frequency elimination is one of the main purposes of our article. Another distinctive feature of this work is the substance detection using both the time interval containing the main THz pulse and the time interval after the main pulse action, despite its strong noise. This is especially important in practice, if the distance between the sample and the THz spectrometer is about 10 m (or longer). In this case, the noise amplitude becomes comparable to the amplitude of the acting THz pulse and it is impossible to accurately determine the time interval containing the reflected from or transmitted through the sample pulse, without increasing the THz pulse power.

We illustrate the choice of optimal spectral resolution by using physical experiments with neutral substances chocolate and cookies. They are easily available food that everybody can buy in the supermarket and in the crowded places—in the shopping mall, at the train station, at the airport or at the stadium. It is in such places that security inspections are often carried out. If the false detection of explosives or other dangerous substances instead of food takes place frequently, the remote screening efficiency will tend to zero. Another reason for this choice is that we show how to detect additional substances (not necessarily harmful) in food, which is important for food quality control. Thus, we consider a typical problem of non-destructive control.

Previously the SDA-method was successfully applied for the identification of substances in transmission and reflection mode in the laboratory conditions (at a distance from the THz emitter to the sample no more than 30 cm) [34,35,36]. In [36] the integral correlation criteria (ICC) were proposed, which were applied for the identification of substances with a complicated surface in the reflection mode. In [37,38,39,40,41] the possibility of identifying neutral and hazardous substances under real conditions (at long distance of about 3.5 m, non-zero relative humidity, in the presence of a strong noise) using the SDA-method together with the ICC was shown. The essential limitations of the standard THz-TDS method for substance detection and identification were also demonstrated. In [42] a computer simulation of a THz pulse interaction with disordered layered structures was made and the occurrence of false absorption frequencies in the spectrum of the signal transmitted through or reflected from such structure was explained.

## 2. Description of the Experimental Setup

The physical experiments with neutral substances (chocolate and cookies) were performed at the Lomonosov Moscow State University (Moscow, Russia) using the THz setup T-SPEC developed by Teravil (Vilnius, Lithuania). It uses a femtosecond fiber laser, which generates laser pulse with average power about 1 W, with 1030 nm centre wavelength, 75 MHz repetition rate, and a pulse duration of 80 fs. Low-temperature grown GaBiAs is used as photoconductor. The spectral range of the spectrometer is 0.1–5.0 THz, signal-to-noise is better than 10^3^:1 (at 2 THz), 10^5^:1 (at 1 THz) and 10^6^:1 (at 0.4 THz), spectral resolution is better than 10 GHz (fast scan), 2.5 GHz (combined mode).

**Figure 1:**
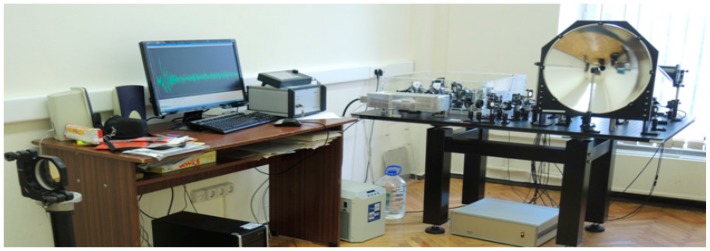
Experimental setup for THz signal measuring at long distance [38].

To focus the THz beam at the remote sample we use a parabolic mirror. Measurements at long distances are additionally equipped with a flat mirror located behind the subject. Therefore, the setup operates in the reflection mode and transmission simultaneously during each measurement. The T-SPEC spectroscopy modules for the reflection and transmission geometry can be found at http://www.ekspla.com. The measurements were carried out under real conditions with a temperature 18 °C and the relative humidity of about 50%. The distance between the parabolic mirror and the object was about 3.5 m. The experimental setup is shown in Figure 1.

An important feature of the setup is the spectral resolution. It is determined by the time interval of a signal measurement, which, as a rule, was equal to 100 ps. Consequently, the minimum frequency difference Δ*ν*, which can be resolved using computer processing of such a signal is 10 GHz, if we do not apply additional efforts to improve the frequency resolution of the spectrometer. The maximum frequency *ν*_max_, which can be detected in the spectrum, depends on the scan step in the time domain Δ*t* and can be increased by computer signal processing.

## 3. SDA-Method and the Integral Correlation Criteria (ICC)

### 3.1. Main Features of the SDA-Method

As is well known, the medium response to a few-cycle THz pulse action (with a duration of about 10 ps) is essentially non-stationary. Analysis of the spectral intensity evolution (the spectral line dynamics) over time at chosen frequency *ν* allows us to obtain much more spectroscopic information about the substance than the signal spectrum analysis only. It gives us an opportunity for more accurate detection of the absorption frequencies from database in the signal under investigation. For the qualitative assessment of this, we propose to use the integral correlation criteria (ICC) [37,38,39,40]. Below we briefly describe the method used.

For detection of the standard substance absorption frequencies in the medium response, we analyze the spectral intensity dynamics. This is achieved by moving the standard signal spectral dynamics at the chosen frequency along the spectral dynamics of the signal under investigation during the chosen time interval. As the standard signal, we use the THz signal transmitted through the sample with the dangerous substance and measured in ideal conditions, or the signal measured in the atmospheric air, taking into account the known absorption frequencies of water vapor. Analyzing the integral correlation between these spectral intensities dynamics, it is possible to arrive at a conclusion about the presence (or absence) of spectral features of the standard substance in the sample under consideration.

### 3.2. Integral Correlation Criteria

To calculate the integral correlation between the spectral line dynamics for the measured signal *E*(*t*) and the standard transmitted signal *s*(*t*) at chosen frequencies, we introduce the following notation: we denote the discrete set of spectral amplitude modulus for the standard transmitted signal *s*(*t*) at chosen frequency *ν*, as $p_{\nu }=\left\{\left|p_{\nu }\left(t_{m}\right)\right|\right\}$, *m* = 1,…,*M*_1_. Let us note that the calculation of the spectral line (or spectral intensity) dynamics *P_ν_*(*t*) at chosen frequency *ν* is described in a number of previous papers, for example, [38,39].

The corresponding set of spectral amplitude modulus of the analyzed THz signal *E*(*t*) at the frequency *ν* is denoted as $P_{\nu }=\left\{\left|P_{\nu }\left(t_{m}\right)\right|\right\}$, *m* = 1,…,*M*_2_, and its part with *M*_1_ components, which begins at time moment *t_n_*, as $P_{\nu }^{\left(n\right)}=\left\{\left|P_{\nu }^{\left(n\right)}\left(t_{n+m}\right)\right|\right\}$, *m* = 1,…,*M*_1_. Here *M*_1_ and *M*_2_ are the numbers of time moments, in which the spectral amplitudes are calculated. They depend on the window length *T* and its shift Δ. We emphasize that to improve the efficiency of detecting the substance presence in the signal under investigation, it is necessary to eliminate the constant component of discrete sets $p_{\nu }=\left\{\left|p_{\nu }\left(t_{m}\right)\right|\right\}$ and $P_{\nu }^{\left(n\right)}=\left\{\left|P_{\nu }^{\left(n\right)}\left(t_{n+m}\right)\right|\right\}$, as it leads to an increase in correlation. Moving the standard set $p_{\nu_{1}}$ along the set under investigation $P_{\nu_{2}}$, we calculate in each time moment *t_n_* the correlation coefficient for two spectral dynamics [38,39]:

(1) $$c_{p,P}(t_{n})=\sum_{m=0}^{M_{1}-1}\left(|p_{\nu_{1}}(t_{m})|-\bar{p_{\nu_{1}}})\cdot (|P_{\nu_{2}}(t_{m+n})|-\bar{P_{\nu_{2}}}\right)/||p_{{}_{\nu_{1}}}-\bar{p_{\nu_{1}}}||\cdot \left||P_{\nu_{2}}^{\left(n\right)}-\bar{P_{\nu_{2}}}|\right|$$

where: $\bar{p_{\nu_{1}}}=\sum_{m=0}^{M_{1}-1}\left|p_{\nu_{1}}(t_{m})\right|/M_{1}$, $\bar{P_{\nu_{2}}}=\sum_{m=0}^{M_{1}-1}\left|P_{\nu_{2}}(t_{m+n})\right|/M_{1}$.

Then, summing these correlation coefficients over time interval [*t*_0_, *t_n_*], we get the following integral characteristic for the detection and identification problem (or integral criterion):

(2) $$C_{p,P}^{}(t_{n})=\sum_{m=0}^{n}\left|c_{p,P}(t_{m})\right|,\;n=0,\ldots ,M_{2}-M_{1}$$

In the present paper, we use the modified criterion (2) taking into account the spectral intensity of each of frequencies *ν*_1_ and *ν*_2_ during the time interval of correlation:

(3) $$CW_{p,P}^{}(t_{n})=\sum_{m=0}^{n}\left|c_{p,P}(t_{m})\right|w_{1}w_{2},\;n=0,\ldots ,M_{2}-M_{1}$$

where $w_{1}=w\left(\left|P\left(\nu_{1}\right)\right|\right)$, $w_{2}=w\left(\left|P\left(\nu_{2}\right)\right|\right)$ are the weight coefficients depending on a spectral intensity of each of the frequencies. For example, they can be chosen as *w*_1_ = 1, *w*_2_ = 1, or $w_{1}=1/\left|P\left(\nu_{1}\right)\right|$, $w_{2}=1/\left|p\left(\nu_{2}\right)\right|$, or $w_{1}=1/{\left(\left|P\left(\nu_{1}\right)\right|\right)}^{2}$, $w_{2}=1/{\left(\left|p\left(\nu_{2}\right)\right|\right)}^{2}$. The last two pairs of weight coefficients take into account the substance absorbance at these frequencies and therefore it is necessary to compute the spectrum brightness of the signal under investigation. Below we use also another modification of ICC (2) [41]:

(4) $$CW1_{p,P}^{}(t_{n})=\sum_{m=0}^{n}\left|c_{p,P}(t_{m})\right|w_{1},\;n=0,\ldots ,M_{2}-M_{1}$$

The main difference between integral criteria (4) and (3) consists in using in (4) the weight coefficient $w_{1}=1/\left|P\left(\nu_{1}\right)\right|$ only. That is, we use only the spectral brightness of the signal from database, and therefore we simplify the algorithm and decrease the influence of random fluctuation on a signal under investigation. One more criterion allows us to assess the similarity (or likeness) of two spectral line dynamics:

(5) $$L_{p,P}^{}(t_{n})=\sum_{m=0}^{n}l_{p,P}^{}(t_{m}),\;n=0,\ldots ,M_{2}-M_{1}$$

where:

(6) $$l_{p,P}(t_{n})=1-\frac{\left||{\left(p_{\nu_{1}}-\bar{p_{\nu_{1}}}\right)}_{N}-{\left(P_{\nu_{2}}{}^{\left(n\right)}-\bar{P_{\nu_{2}}{}^{}}\right)}_{N}|\right|}{\left||{\left(p_{\nu_{1}}-\bar{p_{\nu_{1}}}\right)}_{N}||+||{\left(P_{\nu_{2}}{}^{\left(n\right)}-\bar{P_{\nu_{2}}{}^{}}\right)}_{N}|\right|},\;n=0,\ldots ,M_{2}-M_{1}$$

The subscript *N* indicates that the corresponding variable in (6) must be normalized, for example, in *L*_2_ norm.

It should be emphasized that the correlation coefficients by themselves cannot be used to evaluate the presence of spectral features of desired substances in the signals under investigation due to their strong oscillations at different time moments, which are caused by the modulation of the medium response. Summing them in time allows us to “accumulate” the signal and to suppress fluctuations at different time moments. This procedure is widely used when processing the noisy signals. We stress that each of the integral criteria (2)–(5) is applied in specific situations. For example, ICC (4) is very useful for the detection of the substance absorption frequencies presence in high-noise signals [41].

Note that the frequency *ν*_1_, belonging to a standard substance, is detected in the signal under investigation at the frequency *ν* if the integral criterion, calculated for the frequency pair (*ν*, *ν*_1_), lies above all other lines in the frequency detection range (FDR). As a rule, the boundaries of the FDR are the spectrum extremes closest to the frequency under investigation. Vice versa, the frequency *ν*_1_ is not detected if, at least, one of other lines lies above the line corresponding to the integral criterion, calculated for the pair (*ν*, *ν*_1_), in this FDR.

## 4. The Influence of Spectral Resolution on the Spectral Features of the Signals Chocolate and Cookies

### 4.1. Spectral Resolution of the Signals Chocolate and Cookies Spectra in the Interval t = [0, 110] ps

In this Section we investigate the spectra of the THz signals measured under real conditions in the long time interval *t* > 100 ps, and calculated with different spectral resolutions Δ*ν* = 0.01 THz, 0.04 THz and 0.08 THz. The main question we want to answer is the following: Is it necessary to use the maximal spectral resolution at analyzing such spectra, and how the spectral resolution decreasing influences on the false absorption frequencies presence in the noisy THz signal spectrum? 

Below we show that spectral resolution decreasing for the long-term signal from chocolate allows us to exclude from consideration some false frequencies, which are close to the RDX absorption frequencies or caused by water vapor absorption. However, using this method, it is impossible to eliminate all of them and one needs to use the SDA-method.

**Figure 2:**
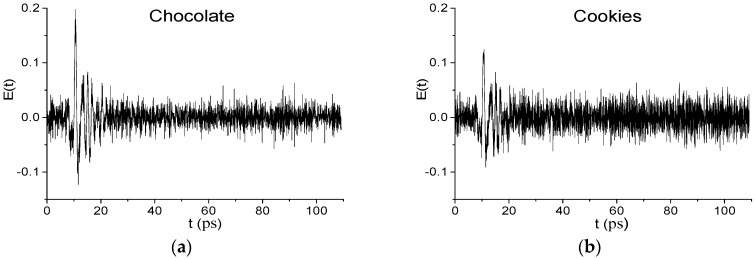
THz signals Chocolate (**a**) and Cookies (**b**) in the time interval *t* = [0, 110] ps.

Figure 2 shows the THz signals Chocolate (a) and Cookies (b) in the time interval *t* = [0, 110] ps. The measurements were performed with a time step Δ*t* = 0.05333 ps. The signals consist of the main pulse (*t* = [0, 25] ps) and the part without pronounced sub-pulses, which follows it (*t* = [25, 110] ps). Reference signal has the same structure. We see that the signals (a), (b) are very noisy. 

The signal-to noise ratio (SNR) can be estimated in the following way. Usually, *SNR* is defined as a ratio of the average of the squares of the amplitudes of the measured THz signal *A_signal_*(*t*) and noise *A_noise_*(*t*) (https://en.wikipedia.org/wiki/Signal-to-noise_ratio):

(7) $$SNR=\bar{A_{signal,n}{}^{2}(t)}/\bar{A_{noise,n}{}^{2}(t)}$$

where $\bar{A_{n}^{2}\left(t\right)}=\overset{N}{\underset{n=1}{\sum }}A_{n}^{2}\left(t\right)/N$, where *N* is a number of time moments in the measurement time interval. As a noise signal *A_noise_*(*t*), we use the reference signal *REF*(*t*) in the time interval *t* = [25, 110] ps length 85 ps, which does not contain the main pulse. As measured THz signals *A_signal_*(*t*) we use Chocolate and Cookies signals’ *E*(*t*) in the time interval *t* = [0, 85] ps of the same length 85 ps, which contains the main pulse. Thus, from (7) we have *SNR* = 2.27 for the signal Chocolate and *SNR* = 1.83 for the signal Cookies.

In practice, it also important to know not only the *SNR*, which is an averaged characteristic of the signal, but also the ratio $CNSR=\;\underset{t\in \left[0,25\right]}{\max}\left|E\left(t\right)\right|/\underset{t\notin \left[0,25\right]}{\max}\left|E\left(t\right)\right|$. Here $\underset{t\in \left[0,25\right]}{\max}\left|E\left(t\right)\right|$ is the maximal amplitude of the noisy THz signal *E*(*t*) in the time interval *t* = [0, 25] ps containing the useful signal, and $\underset{t\notin \left[0,25\right]}{\max}\left|E\left(t\right)\right|$ is the maximal amplitude of the noisy part of this signal in the time interval *t* = [25, 110] ps, which does not contain the main pulse. Thus, *CSNR_Chocolate_* = 3.1434, *CSNR_Cookies_* = 1.85.

**Figure 3:**
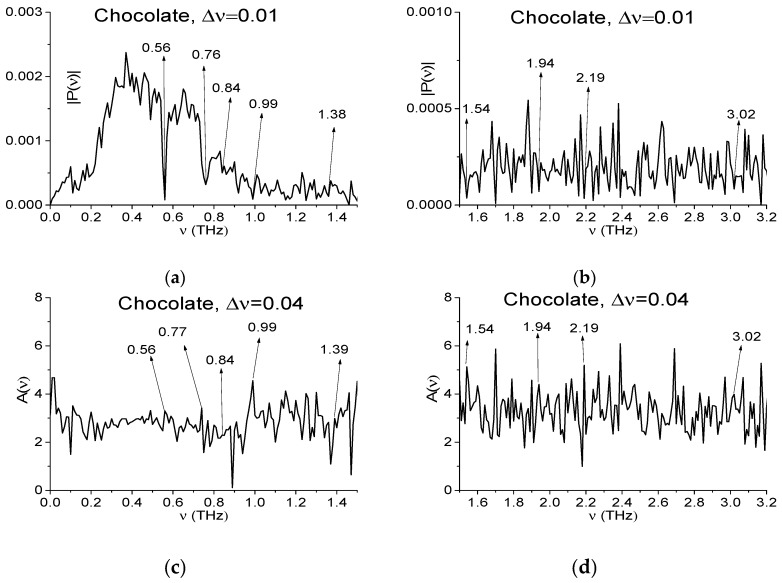

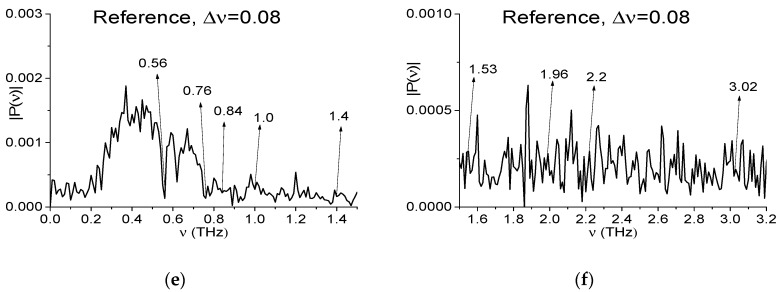
Fourier spectrum (**a**,**b**), absorbance (**c**,**d**) of the signal Chocolate and Reference spectrum (**e**,**f**) in the frequency ranges *ν* = [0, 1.5] THz (**a**,**c**,**e**) and [1.5, 3.2] THz (**b**,**d**,**f**) calculated with spectral resolution Δ*ν* = 0.01 THz.

Consequently, the Fourier spectrum (a), (b), absorbance *A*(*ν*) (c), (d) of the Chocolate signal as well as Reference spectrum (e), (f), which are presented in Figure 3 in the frequency ranges *ν* = [0, 1.5] THz (a), (c), (e) and [1.5, 3.2] THz (b), (d), (f) are strongly and randomly modulated. Here:

(8) $$A(\nu )=-\log_{10}(|P(\nu )|/|P_{REF}(\nu )|)$$

|P(ν)|, $\left|P_{REF}\left(\nu \right)\right|$ are the modules of the signal spectral amplitude and corresponding value for Reference. All spectral functions in (a)–(f) are calculated with spectral resolution Δ*ν* = 0.01 THz and contain a great number of peaks and minima.

In the experiments carried out the hazardous substances are absent in the samples with chocolate and cookies. However, in Fourier spectra of these signals one can observe the minima (and the corresponding peaks in absorbance), which are close to the absorption frequencies of many hazardous substances. For example, the explosive RDX has the following absorption frequencies: ν = 0.82, 1.05, 1.36, 1.54, 1.95, 2.19, 3.0 THz [3,4,5]. In Figure 3 the minima (a), (b) and peaks (c), (d) at frequencies ν = 0.84, 0.99, 1.38 (1.39 in (c)), 1.54, 1.94, 2.19, 3.02 THz, which are close to them, are marked. At the same time, in the Reference spectrum (e), (f) one can see minima at the frequencies ν = 0.84, 1.0, 1.4, 1.53, 1.96, 2.2, 3.02 THz. Some of them may be caused by water vapor absorption. The corresponding absorption frequencies are given in [16], and they are equal to: ν = 0.56, 0.75, 0.99, 1.11, 1.16, 1.21, 1.23, 1.32, 1.41, 1.6, 1.67, 1.72, 1.76, 1.79, 1.88, 1.92 THz. Other minima in (a), (b) (and peaks in (c), (d)) may be caused by the influence of environment or by small-scale modulation of the medium response or by measurement setup. Note that one can find among them minima close to the absorption frequencies of many dangerous and neutral substances. Therefore, the signal spectrum cannot provide all the necessary fingerprints for true identification of a substance.

**Figure 4:**
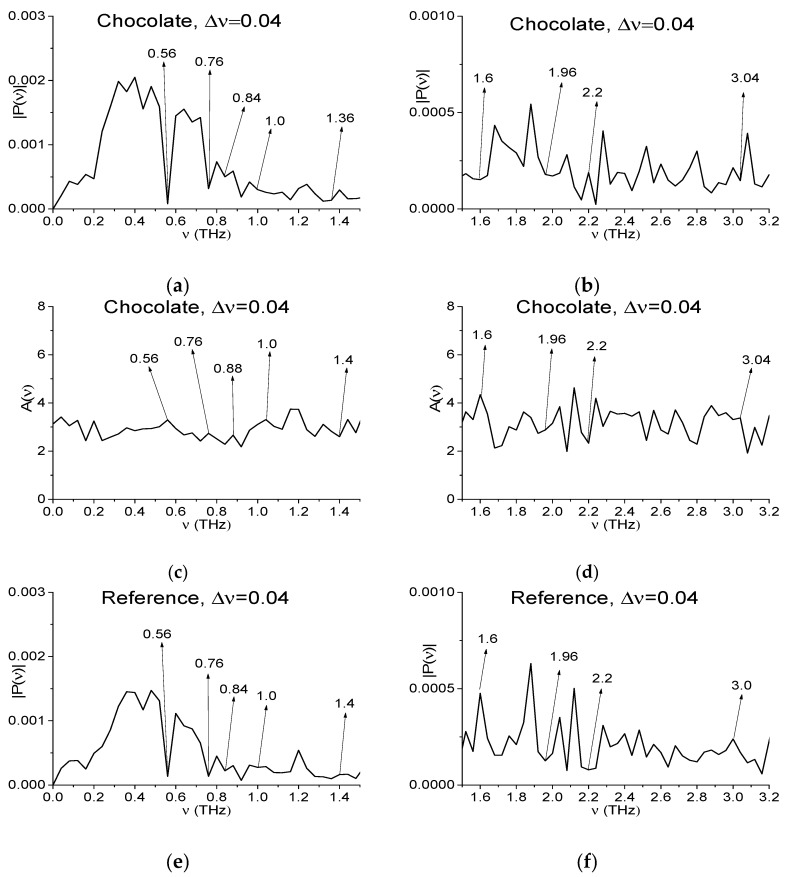
Fourier spectrum of the signal Chocolate (**a**,**b**), absorbance (**c**,**d**) and Reference spectrum (**e**,**f**) in the frequency ranges *ν* = [0, 1.5] THz (**a**,**c**,**e**) and [1.5, 3.2] THz (**b**,**d**,**f**) calculated with spectral resolution Δ*ν* = 0.04 THz.

One of the ways to decrease the number of such minima consists in decreasing the spectral resolution at analyzing the signal spectrum (and absorbance). In Figure 4 we show the same spectral characteristics as in Figure 3a–f in the frequency ranges *ν* = [0, 1.5] THz (a), (c), (e) and [1.5, 3.2] THz (b), (d), (f) calculated with spectral resolution Δ*ν* = 0.04 THz.

In order to obtain the required spectral characteristics, we use the following procedure. Consider the Chocolate signal spectrum shown in Figure 3a,b as an example. Let $\left|P\left(\nu_{i}\right)\right|$, *i* = 1,2,…,*M_ν_*+1 be the set of the signal spectral amplitude modules, computed for the frequencies *ν_i_* = (*i* – 1)·*h_ν_*, where the *h_ν_* = 0.01 THz is a step in the frequency domain *ν* = [0,Lν] THz, *M_ν_* = *L_ν_*/*h_ν_* is a number of mesh nodes in this frequency domain. Obviously, in this case the spectral resolution Δ*ν* is equal to the frequency domain step *h_ν_* = 0.01 THz. To decrease the spectral resolution from Δ*ν* = 0.01 THz up to Δ*ν* = 0.04 THz, we choose in the set *ν_i_* the nodes $\nu_{i}^{0.04}=4\left(i-1\right)\cdot h_{\nu }=\left(i-1\right)\cdot 4h_{\nu }$, where 4*h_ν_* = 0.04 THz, *i* = 1,2,…,$\mathrm{int}\left(\left(M_{\nu }+1\right)/4\right)$. Here the expression $\mathrm{int}\left(\left(M_{\nu }+1\right)/4\right)$ means an integer part of the number $\left(M_{\nu }+1\right)/4$. Each frequency $\nu_{i}^{0.04}$ corresponds to the spectral amplitude modules $\left|P(\nu_{i}^{0.04})\right|,$ *i* = 1,2,…,$\mathrm{int}\left(\left(M_{\nu }+1\right)/4\right)$. In order to depict the Chocolate spectrum with the spectral resolution Δ*ν* = 0.04 THz, we use the formula for a straight line *y*(*x*) passing through the points (*x_i_*,*y_i_*) = $\left(\nu_{i}^{0.04},\left|P\left(\nu_{i}^{0.04}\right)|\right|\right)$ and (*x_i+_*_1_,*y_i+_*_1_) = $\left(\nu_{i+1}^{0.04},\left|P\left(\nu_{i+1}^{0.04}\right)|\right|\right)$:

(9) $$\frac{y(x)-y_{i}}{y_{i+1}-y_{i}}=\frac{x-x_{i}}{x_{i+1}-x_{i}},\;i=1{,}_{}2,\ldots ,\;\mathrm{int}((M_{\nu }+1)/4)-1$$

From (9) we have the well-known expression for a straight line *y*(*x*):

(10) $$y(x)=k\cdot x+b,\;k=\frac{y_{i+1}-y_{i}}{x_{i+1}-x_{i}},\;b=y_{i}-x_{i}\;\frac{y_{i+1}-y_{i}}{x_{i+1}-x_{i}},i=1{,}_{}2,\ldots ,\;\mathrm{int}((M_{\nu }+1)/4)-1$$

Note that in this procedure, the cos- and sin- Fourier coefficients corresponding to the frequency domain points $\nu_{i}^{0.04}$ are preserved and they can be used for the inverse Fourier transform. The spectrum of the Reference signal with spectral resolution Δ*ν* = 0.04 THz (Figure 4e,f) is computed in the same way. The corresponding Absorbance (Figure 4c,d) is computed using expression (8).

Consequently, one can see the significant minima number decreasing in (a), (b), (e), (f) and peaks in (c), (d). There is no more minima in (a), (b) (peaks in (c), (d)) at frequencies ν = 1.0, 2.2 THz, which are close to the RDX absorption frequencies. At the same time, there are still minima in (a), (b) (peaks in (c), (d)) at the frequencies ν = 0.84 (0.88 in (c)), 1.36, 3.04 THz. The extremes at the frequencies ν = 0.56, 0.76 THz also are saved in all figures (a)–(f). Thus, they are due to atmospheric water vapor absorption because they are also present in the Reference spectrum (e).

**Figure 5:**
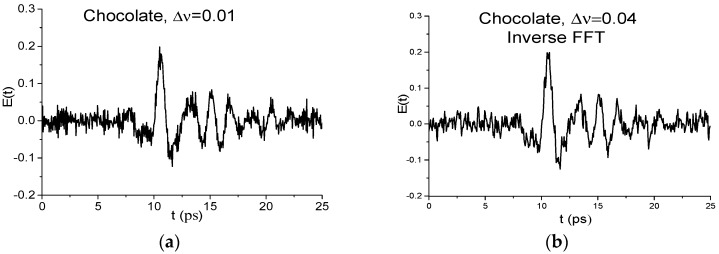
The main pulse of the measured THz signal Chocolate (**a**) and recovered with inverse Fourier transform signal Chocololate_0.04 (**b**) in the time interval *t* = [0, 25] ps.

The next question we want to answer is the following: What is the impact of decreasing spectral resolution on the SNR for the THz signals under investigation? Figure 5 presents the main pulse of the measured THz signal Chocolate (a) and the restored with inverse Fourier transform signal Chocolate_0.04 (b) corresponding to the spectrum in Figure 4a,b. The number of small-scale oscillations in (b) is decreased in comparison with (a). However, in the structure of the signal Chocolate_0.04 it is clearly seen that the maximal amplitude oscillations are due to the lower frequencies presence in the signal. Therefore, when decreasing the spectral resolution for the given THz signals, SNR and CSNR practically do not change. Nevertheless, below we show that decreasing spectral resolution in the case of measurements under real conditions may be the alternative to the noise reduction methods.

**Figure 6:**
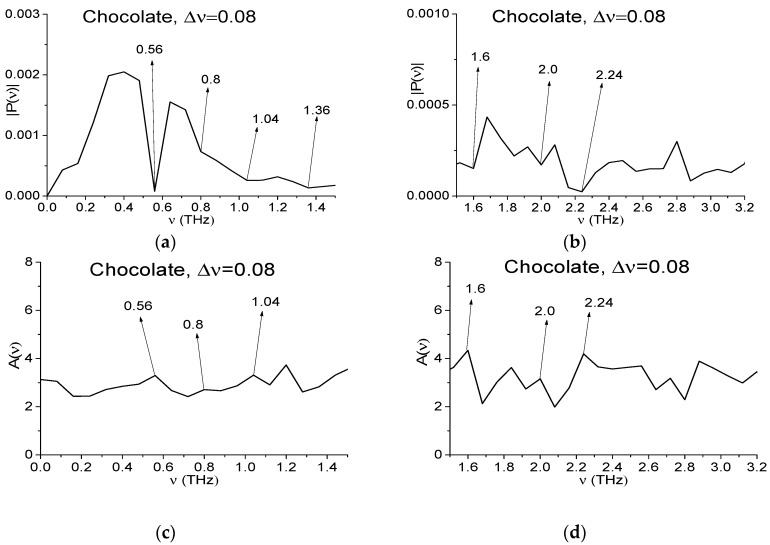

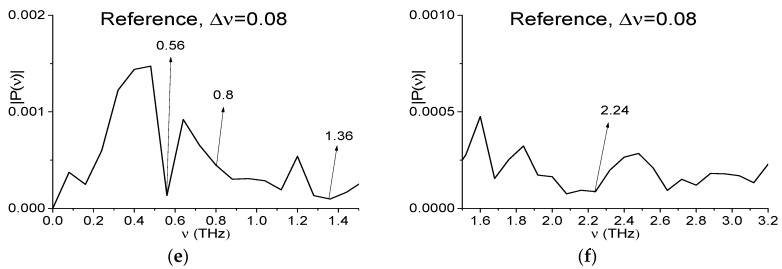
Fourier spectrum of the signal Chocolate (**a**,**b**), absorbance (**c**,**d**) and Reference spectrum (**e**,**f**) in the frequency ranges *ν* = [0, 1.5] THz (**a**,**c**,**e**) and [1.5, 3.2] THz (**b**,**d**,**f**), the corresponding spectral resolution is equal to Δ*ν* = 0.08 THz.

In Figure 6 we decrease the spectral resolution for the spectral characteristics of the Chocolate signal and Reference up to the value Δ*ν* = 0.08 THz. The decreasing procedure is described above for the spectral resolution Δ*ν* = 0.04 THz, see Equations (9) and (10). The corresponding frequency ranges are equal to *ν* = [0, 1.5] THz (a), (c), (e) and [1.5, 3.2] THz (b), (d), (f).

In (a), (b) minima are present, which are close to the RDX absorption frequencies ν = 0.8 (inflection point), 1.36, 1.6, 2.0, 2.24 THz, the corresponding peaks in (c), (d) are ν = 0.8, 1.04, 1.6, 2.0, 2.24 THz. At the same time in (e), (f) the minima take place at ν = 0.84, 1.36, 2.24 THz. The extreme at frequency ν = 0.56 THz remains in all figures (a)–(f). Above we showed that it is caused by water vapor absorption of THz radiation. As to the absence of the spectrum minimum at frequency close to ν = 0.76 THz, then its absence in the spectrum may be due to the fact that a half-width of this water vapor absorption line is less than a spectral resolution Δ*ν* = 0.08 THz. The extremes at frequencies ν = 1.36, 2.24 THz may be caused by the influence of environment.

One can see from Figure 6a that the minima at frequency ν = 0.8 THz in the Chocolate spectrum is absent. This means that the half-width of the Chocolate absorption line at the frequency ν = 0.84 THz is less than 0.04 THz, which does not correspond to the RDX absorption spectral line. In [3,4,5] it was shown that RDX absorption line half-width at the frequency ν = 0.82 THz is not less than 0.1 THz. Therefore, if RDX is present in the chocolate, then in the main pulse spectrum Figure 6a a minimum at the frequency close to the value ν = 0.82 THz would have to be (at ν = 0.8 THz due to spectral resolution Δ*ν* = 0.08 THz). Thus, the spectrum minimum in Figure 3a at the frequency ν = 0.84 THz is caused by small-scale modulation of the signal due to external factors but not the RDX presence. Nevertheless, we see in Figure 6c a weak maximum of the absorbance. A detailed discussion of this phenomenon will be continued in Section 4.2.

It is worth noting when decreasing the spectral resolution up to Δ*ν* = 0.08 THz, the inverse Fourier transform restores the Chocolate signal in the time interval *t* = [0, 12.5] ps with length less than the main pulse duration. This means the loss of the part of important information about the spectral characteristics of the investigated signal, which is contained in the main pulse.

Therefore, the spectral resolution decreasing for the long-term signal Chocolate allows us to exclude from consideration the frequencies ν = 0.84, 0.99 THz, which are close to those of RDX and to confirm that the spectral minima at the frequencies ν = 0.56, 0.76 THz are caused by the water vapor absorption. However, one can see that it is impossible to eliminate all false frequencies only analyzing the spectrum of a long-term THz signal and its Reference with different spectral resolution.

Below, we study the spectral properties of the signal Chocolate in various time intervals of smaller duration.

### 4.2. Spectral Resolution Influence on the Spectra of the Chocolate and Cookies Main Pulses Calculated in the Time Interval t = [0, 25] ps

In this Section, we investigate the spectral resolution influence on the main pulse spectra of the signals Chocolate and Cookies. The main pulses are located in the short time interval *t* = [0, 25] ps, so, their spectra contain much less of the minima, caused by the influence of environment, in comparison with the spectra obtained in the time interval *t* = [0, 110] ps*.*

As in the Section 4.1, the Chocolate spectrum analysis in the time interval *t* = [0, 25] ps at decreasing spectral resolution allows us to remove some false absorption frequencies (close to those of RDX or water vapor) from consideration. At the same time, the part of them still takes place in the spectrum obtained with low spectral resolution. Consequently, one has to use a more effective tool for the signal treatment. Such a tool may be the ICC.

The main pulse of the Chocolate signal is depicted in Figure 5a. The signal Cookies main pulse has same structure.

**Figure 7:**
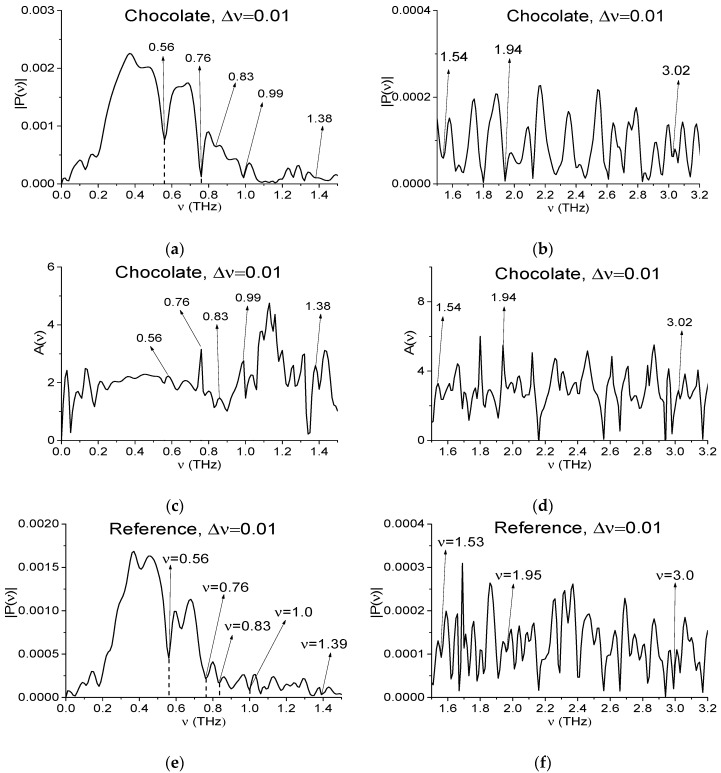
The signal Chocolate main pulse Fourier spectrum (**a**,**b**); the Reference spectrum (**e**,**f**) and the substance absorbance (**c**,**d**) in the frequency ranges *ν* = [0, 1.5] THz (**a**,**c**,**e**) and [1.5, 3.2] THz (**b**,**d**,**f**); the corresponding spectral resolution is equal to Δ*ν* = 0.01 THz.

Figure 7 shows the main pulse Fourier spectrum (*t* = [0, 25] ps) for the Chocolate signal in the frequency ranges *ν* = [0, 1.5] THz (a) and [1.5, 3.2] THz (b) and the corresponding values of the absorbance *A*(*ν*) (c), (d). In Figure 7e,f the Reference spectrum is presented in the same frequency ranges. The spectral resolution in (a)–(d) is equal to Δ*ν* = 0.01 THz. In order to get such resolution we removed a part of the measured signal corresponding to the time *t* > 25 ps, and then added zeroes to the signal up to the duration *t* = 100 ps.

As in Section 4.1, the Fourier spectrum minima for the Chocolate main pulse corresponding to the frequencies *ν* = 0.56 and 0.76 THz (a) are caused by atmospheric water vapor absorption. The peaks at these frequencies in Figure 7c and pronounced minima in the Reference spectrum depicted in Figure 7c confirms this conclusion.

In Figure 7a–d one can also see the minima (a), (b) and peaks (c), (d) at the frequencies *ν* = 0.83, 0.99 (1.0 in (e)), 1.38, 1.53, 1.67, 1.94, 3.02 THz, which are close to RDX absorption frequencies. It should be noted that in the Reference spectrum (e), (f) there are minima at the same or close frequencies, which are caused by water vapor absorption or environment influence.

In the Chocolate signal spectrum, one can also find the minima at the absorption frequencies of other hazardous substances. So, the illicit drugs MA (methamphetamine) and MDMA (methylenedioxymethamphetamine, or “ecstasy”) have absorption frequencies ν = 1.23, 1.67, 1.86 THz (MA) and ν = 1.2, 1.9 THz (MDMA) in the frequency range *ν* < 2.5 [43]. In [34,44] when studying the spectral properties of MA and MDMA, the following absorption frequencies were found: ν = 1.25, 1.65, 1.85, 2.65 THz (MA) and ν = 1.25, 1.95 THz (MDMA). They are in good agreement with those given in [34,45]. The part of the frequencies close to them also are present in the Fourier spectrum (a), (b) and absorbance (c), (d) of the Chocolate signal.

Similar results occur for the Cookies signal, as in its Fourier spectrum there are the minima (peaks in the absorbance) at the frequencies close to the absorption frequencies of RDX, MA and MDMA. These examples demonstrate the fundamental limitations of the standard THz TDS method under real conditions, because it can give a lot false information about the presence of hazardous substances in neutral ones.

**Figure 8:**
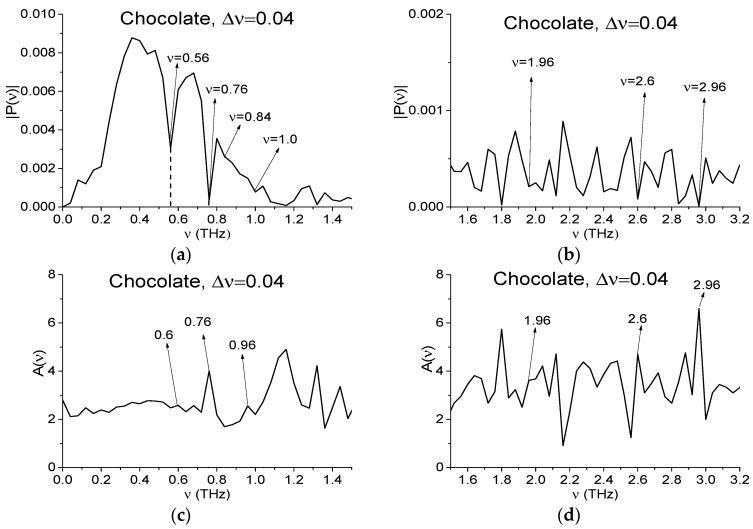

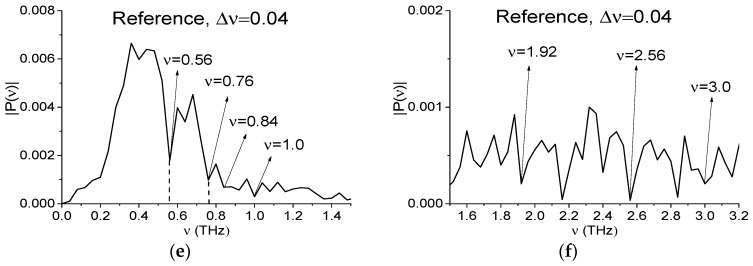
The Chocolate main pulse Fourier spectrum (**a**,**b**), Reference spectrum (**e**,**f**) and absorbance (**c**,**d**) in the frequency ranges ν = [0, 1.5] THz (**a**,**c**,**e**), [1.5, 3.2] THz (**b**,**d**,**f**) calculated with spectral resolution Δ*ν* = 0.04 THz.

Figure 8 shows the Fourier spectrum of the signal Chocolate main pulse (a), (b), absorbance (c), (d) and Reference Fourier spectrum (e), (f) in the frequency ranges ν = [0, 1.5] THz (a), (c), (e), [1.5, 3.2] THz (b), (d), (f) calculated in the time interval *t* = [0, 25] ps with a decreased spectral resolution Δ*ν* = 0.04 THz.

We see that the spectral minima in (a), (b) corresponding to RDX absorption frequencies ν = 0.84, 1.4 THz, are not preserved in the spectrum (either peaks in absorbance, they are not shown). However, at the frequency ν = 0.84 THz an inflection point of the spectrum takes place. On the other hand, one can see pronounced spectral minima at the frequencies ν = 1.0, 3.0 THz in the Reference spectrum (c), (d), which also takes place in (a), (b) (3.04 in (b)). That means that the minima in (a), (b) at these frequencies can be masked or caused by water vapor absorption. The number of the minima in (a), (b), corresponding to MA and MDMA absorption frequencies, also decreases with the spectral resolution decreasing. But the spectral minimum (peak in (d)) at the frequency ν = 2.6 THz, close to MA absorption frequency ν = 2.65 THz still takes place in (b). Since the spectral minimum at this frequency in Reference spectrum (f) is absent (there is a minimum at ν = 2.56 THz) it is still necessary to check this minimum to be the MA absorption frequency.

**Figure 9:**
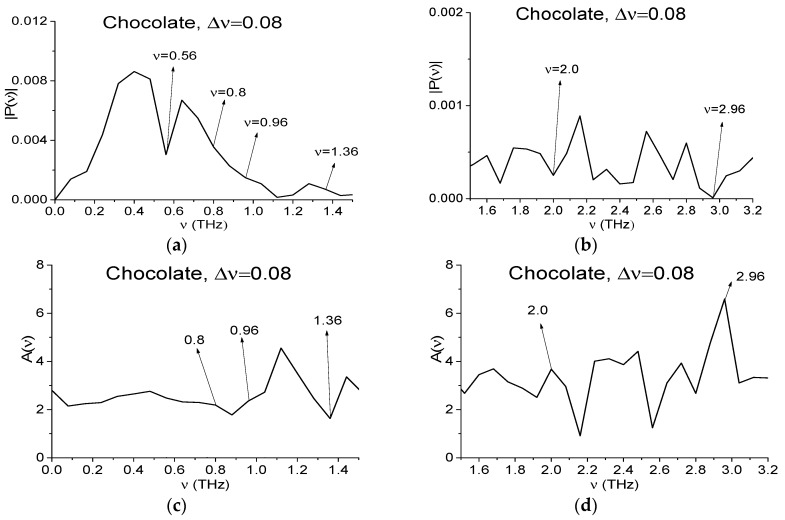

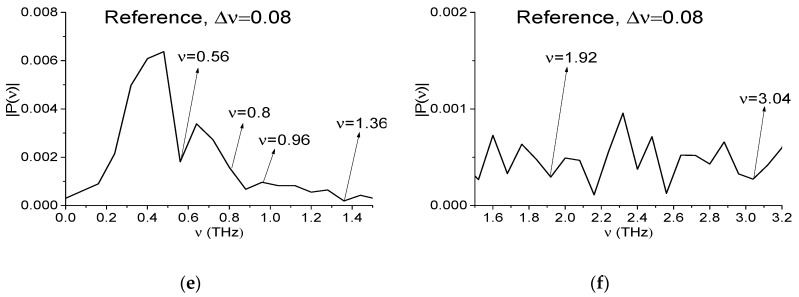
The Chocolate main pulse Fourier spectrum (**a**,**b**), Reference spectrum (**e**,**f**) and absorbance (**c**,**d**) in the frequency ranges ν = [0, 1.5] THz (**a**,**c**,**e**), [1.5, 3.2] THz (**b**,**d**,**f**) calculated with spectral resolution Δ*ν* = 0.08 THz.

In Figure 9 the Chocolate signal main pulse Fourier spectrum (a), (b), absorbance (c), (d) and the Reference spectrum (e), (f) are depicted in the same frequency ranges ν = [0, 1.5] THz (a), (c), (e), [1.5, 3.2] THz (b), (d), (f). They are calculated in the time interval *t* = [0, 25] ps with a spectral resolution Δ*ν* = 0.08 THz. The spectral minima in (a), (b) (the peaks in absorbance (c), (d)) corresponding to RDX absorption frequencies ν = 0.8, 1.0, 1.36 THz, are absent in the spectrum. At the frequency ν = 0.8 THz there is not an inflection point either. On the other hand, one can see pronounced spectral minima at the frequencies ν = 2.0, 2.96 THz in (a), (b) (peaks in (c), (d)) and the spectral minima in (e), (f) at frequencies ν = 1.92, 3.04 THz close to them. That means that the spectral minima in (a), (b) at these frequencies can be caused by influence of environment. The spectral minimum at the frequency ν = 2.64 THz, close to MA absorption frequency ν = 2.65 THz, is absent in (b), the corresponding peak in (d) is also absent. The minimum at this frequency in the Reference spectrum (f) is absent.

Therefore, the Chocolate spectrum analysis in the time interval *t* = [0, 25] ps with decreasing spectral resolution only gives us the opportunity to remove some false absorption frequencies (close to those of RDX or water vapor) from consideration. At the same time, one cannot unambiguously determine the absence of the dangerous substance absorption frequencies in the signal under investigation, because the part of them still occurs in the spectrum.

**Figure 10:**
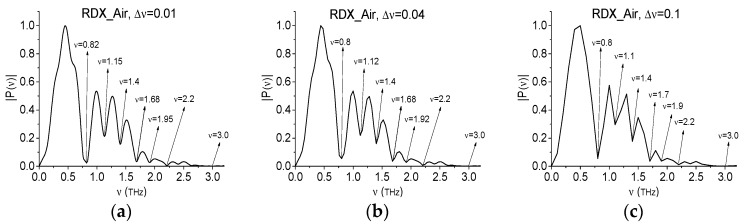

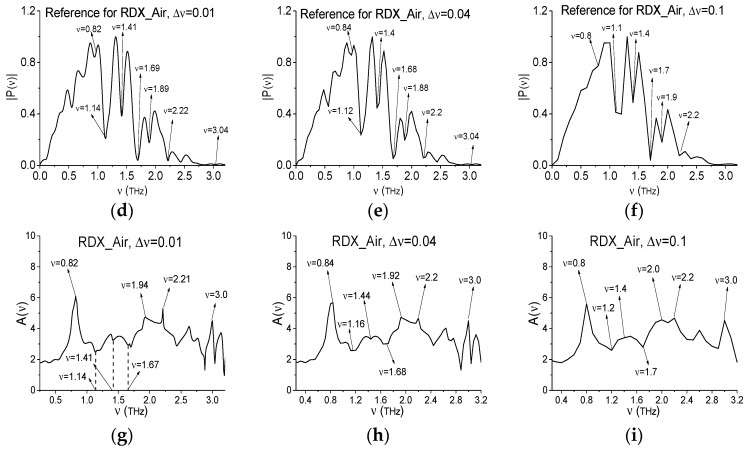
The standard signal RDX_Air Fourier spectra (**a**–**c**), Reference spectrum (**d**–**f**), absorbance (**g**,**h**) calculated with the spectral resolution Δ*ν* = 0.01 THz (**a**,**d**,**g**), 0.04 THz (**b**,**e**,**h**), 0.1 THz (**c**,**f**,**i**).

For the purpose of the ICC using, we will use the THz signal transmitted through the pellet containing 10% RDX and 90% polyethylene in the air (we denote it RDX_Air for brevity) as a standard signal. The signal was measured in a short time interval 0 < *t* < 10 ps at 22 °C and a relative humidity of about 50% at the Center for Terahertz Research, Rensselaer Polytechnic Institute (Troy, NY, USA). Figure 10 shows the normalized Fourier spectra of the signal RDX Air and Reference spectrum (d)–(f) calculated with the frequency resolution (the feasibility of this is discussed below) Δ*ν* = 0.01 (a), (d), 0.04 (b), (e), 0.1 THz (c), (f). Taking into account each spectral resolution, we see that the spectral minima are in good agreement with those given in [3,4,5]. The absorption peaks position in (g)–(i) is in a good agreement with the corresponding minima position in (a)–(c).

In [36] it was shown that the appearance of spectral minima at the frequencies ν = 1.15, 1.4, 1.68 THz in the Fourier spectrum (a) for the standard signal RDX_Air is caused by water vapor absorption (see above its absorption frequencies obtained in [16]). The presence of minima in the Reference spectra (d)–(f) and the absence of peaks in the absorbance (g,h) at the spectral resolution decreasing confirms this conclusion. In turn, the Fourier spectrum minima for the signal RDX_Air at the frequencies ν = 0.82, 1.95, 2.2 and 3.0 THz can be used for the RDX detection in the sample under investigation. Note that in the Chocolate signal spectrum (Figure 7b) there are no minima at the RDX absorption frequencies ν = 2.2, 3.0 THz. However, the maxima or minima at these frequencies are also absent in the absorbance (Figure 7d), which does not allow to exclude these frequencies from consideration on the subject of their belonging to substance RDX. So, we will use ICC.

**Figure 11:**
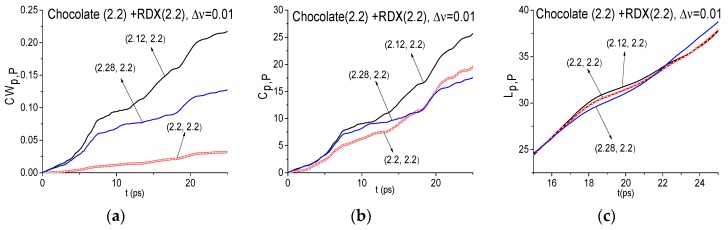

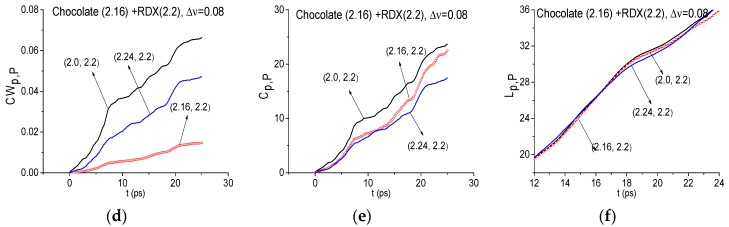
ICC’s *CW_p,P_* (**a**), *C_p,P_* (**b**) and *L_p,P_* (**c**), calculated at the frequency ν = 2.2 THz for signal Chocolate and the standard RDX_Air signal with the spectral resolution Δ*ν* = 0.01, 0.04 THz (**a**–**c**), 0.08 THz (**d**–**f**).

Figure 11 shows the ICC’s *CW_p,P_* (a), *C_p,P_* (b) and *L_p,P_* (c) evolution, calculated for the frequency ν = 2.2 THz with the spectral resolution Δ*ν* = 0.01 THz. Here the FDR is ν = [2.12, 2.28] THz, where the frequencies ν = 2.12, 2.28 THz are the local minima of the signal Chocolate spectrum closest to the investigated frequency ν = 2.2 THz. ICC’s (a)–(c) lie below other lines from the FRD in the entire time interval *t* = [0, 25] ps. As the integral criterion lines *L_p,P_* are very close to each other, in Figure 11c they are shown in the decreased time interval *t* = [17, 25] ps, where one can clearly see that the line *L_p,P_* calculated for the frequency ν = 2.2 THz is not the topmost. Consequently, the ICC’s (a)–(c) do not detect the frequency ν = 2.2 THz as the absorption frequency of a substance RDX. Similarly, the frequency ν = 3.0 THz was not detected as the RDX absorption frequency either.

It is interesting to note that for the spectral resolution Δ*ν* = 0.04 THz the evolution of ICC’s *CW_p,P_*, *C_p,P_* and *L_p,P_* for the frequency ν = 2.2 THz is the same as for the resolution Δ*ν* = 0.01 THz with the same FDR ν = [2.12, 2.28] THz. It means that the half-width of the corresponding spectral line is not less than Δ*ν* = 0.04 THz. Thus, we do not show these ICC’s in Figure 10. If we decrease the spectral resolution up to Δ*ν* = 0.08 THz then this absorption frequency is shifted to ν = 2.16 THz (Figure 9b). Because this frequency is close to the RDX absorption frequency ν = 2.2 THz, in (d)–(f) the evolution of ICC’s *CW_p,P_*, *C_p,P_* and *L_p,P_* is presented calculated at the frequency ν = 2.16 THz with the spectral resolution Δ*ν* = 0.08 THz and with the FDR ν = [2.0, 2.24] THz. We see that the ICC’s (d)–(f) do not detect the frequency ν = 2.16 THz as the RDX absorption frequency in the Chocolate signal. It is of interest that the detection contrast in (d)–(f) is practically the same as for the spectral resolution Δ*ν* = 0.01, 0.04 THz. Thus, the decreasing spectral resolution does not degrade the detection contrast in comparison with (a)–(c).

ICC allow us to show, in many cases, the absence of the standard substance spectral features, even if in the spectrum of the signal under investigation there is a minimum at the frequency, coinciding or close to the standard absorption frequency. However, one can observe a very complicated situation at these criteria using without changing the spectral resolution and taking into account the bandwidth of the standard substance absorption frequencies. To illustrate this, let us consider the frequency ν = 0.83 THz, which corresponds to the Chocolate signal spectrum minimum in the time interval *t* = [0, 25] ps (Figure 7a). We emphasize that in the Reference spectrum one can also observe the minimum at the frequency ν = 0.83 THz (Figure 7c), caused by the influence of some air gas or small-scale modulation of the signal due to noise. We stress that the water vapor absorption is absent at this frequency [16].

**Figure 12:**
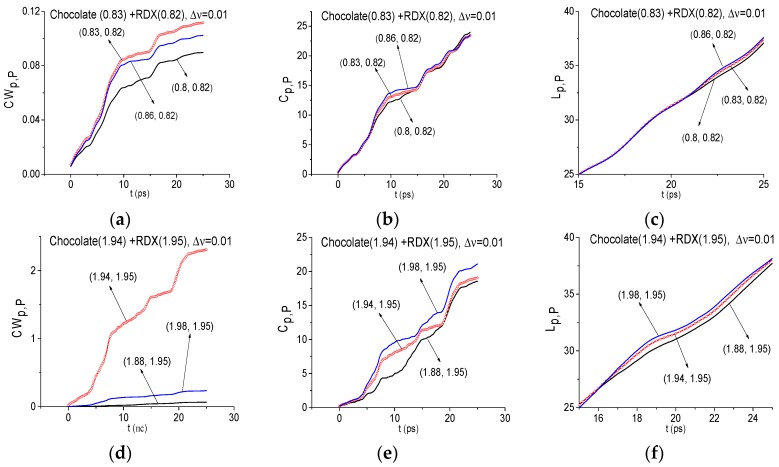
ICC’s *CW_p,P_* (**a**,**d**) *C_p,P_* (**b**,**e**) and *L_p,P_* (**c**,**f**), calculated for frequencies *ν* = 0.83 THz (**a**–**c**), 1.94 THz (**d**–**f**) with the spectral resolution Δ*ν* = 0.01 THz for signal Chocolate and the standard RDX_Air signal.

In Figure 12 the ICC’s *CW_p,P_* (a), *C_p,P_* (b) and *L_p,P_* (c) calculated for the frequency ν = 0.83 THz with the spectral resolution Δ*ν* = 0.01 THz are shown for the FDR ν = [0.8, 0.86] THz. It can be seen, the values of ICC *CW_p,P_* calculated for the frequency ν = 0.83 THz (a), lie above the others. However, as we mentioned above, in the Reference spectrum there is also a minimum at this frequency. Thus, the topmost position of the line corresponding to the frequency ν = 0.83 THz in (a) may be caused by influence of environment or small-scale modulation. At the same time, we see that the ICC’s *C_p,P_* (b) and *L_p,P_* (c) show the RDX spectral features absence in the Chocolate signal. For greater clarity, the evolution of *L_p,P_* is shown in the decreased time interval *t* = [17, 25] ps due to the close location of these lines in the interval *t* = [0, 25] ps. Thus, in this situation, it is impossible to make the unambiguous conclusion about this frequency and it is necessary to make an additional investigation.

Similar results were obtained for the frequency ν = 1.94 THz, which is the Chocolate signal spectrum minimum in the time interval *t* = [0, 25] ps and is close (with an accuracy up to Δ*ν* = 0.01 THz) to the absorption frequency ν = 1.95 THz of the standard RDX_Air signal. In the Reference spectrum (Figure 7f) the minimum is also observed close to the frequency ν = 1.95 THz due to the influence of the environment or small-scale modulation of the measured signal. This minimum is close to water vapor absorption frequency ν = 1.92 [16]. This fact may explain that the values of ICC *CW_p,P_* calculated for the frequency ν = 1.94 THz (d), lie above other lines. However, two other ICC’s *C_p,P_* (e) and *L_p,P_* (f) do not show the presence of RDX spectral feature at the frequency ν = 1.94 THz in the signal Chocolate.

Thus, it is not clear whether the frequencies ν = 0.83 THz and 1.94 THz belong to RDX or not and it is necessary to provide additional investigation. As we demonstrate in Section 4.1, there is another way to show that the frequency ν = 0.83 THz is not the RDX absorption frequency in the Chocolate signal. For this purpose, it is necessary to decrease the spectral resolution when calculating the Fourier spectrum of the signal under investigation. If the spectral minimum disappears in this spectrum, but it is kept in the standard RDX_Air signal spectrum, it means that it was caused by the small-scale modulation of the signal.

**Figure 13:**
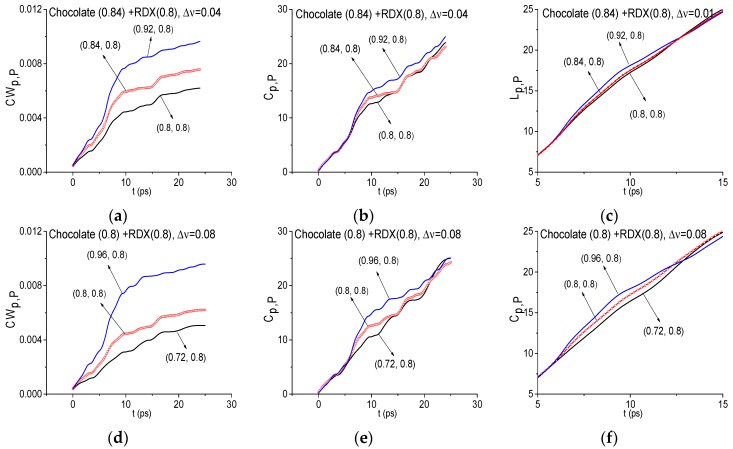
Time-dependent ICC’s *CW_p,P_* (**a**,**d**), *C_p,P_* (**b**,**e**) and *L_p,P_* (**c**,**f**) calculated for the frequency *ν* = 0.84 THz (**a**–**c**), 0.8 THz (**d**–**f**) with the spectral resolution Δ*ν* = 0.04 THz (**a**–**c**), 0.08 THz (**d**–**f**) for signal Chocolate and the standard RDX_Air signal.

The similar possibility for the false frequency elimination gives us the ICC analysis with decreased spectral resolution. Figure 13 shows the ICC’s *CW_p,P_* (a), (d), *C_p,P_* (b), (e) and *L_p,P_* (c), (f) evolution calculated for the frequency ν = 0.84 THz (a)–(c), 0.8 THz (d)–(f) with the spectral resolution Δ*ν* = 0.04 THz (a)–(c), 0.08 THz (d)–(f). The corresponding FDRs are chosen as ν = [0.8, 0.92] THz (a)–(c), [0.8, 0.96] THz and they are increased in comparison with Figure 12. The *L_p,P_* changing (c) (f) is also shown in the decreased time interval *t* = [5, 15] ps due to the close location of these spectral lines in the time interval *t* = [0, 25] ps.

It is clearly seen that all ICC’s show the RDX spectral features absence in the Chocolate signal. We see also that with the spectral resolution decreasing from Δ*ν* = 0.01 THz to Δ*ν* = 0.04, 0.08 THz the detection contrast at using the ICC increases. In the same way it is possible to show that the frequency ν = 1.94 THz is not the RDX absorption frequency in the Chocolate signal.

Thus, for a highly noisy THz signal the spectral resolution increasingly leads to observation of the THz pulse spectrum modulation. Changing the spectral resolution is an effective tool for elimination of the false absorption frequencies. The selection is made using the measured signal spectrum and ICC. We demonstrate this on the basis of the RDX absorption frequencies analysis in the Chocolate signal. For this signal, the spectral resolution decreasing from Δ*ν* = 0.01 THz to Δ*ν* = 0.04, 0.08 THz allows us to eliminate from consideration the false RDX absorption frequencies, which were initially observed in the signal Chocolate spectrum calculated with spectral resolution Δ*ν* = 0.01 THz. Therefore, to identify the substance with high probability, it is necessary to take into account the absorption line bandwidth.

We show that the detection contrast increases for the ICC’s *CW_p,P_*, *C_p,P_* and *L_p,P_* at the spectral resolution decreasing from Δ*ν* = 0.04 THz to Δ*ν* = 0.08 THz. The absence of other substances spectral features for example MA and MDMA, can be shown in a similar way. The similar results are valid for the Cookies signal spectrum.

### 4.3. The Spectral Resolution Influence on the Signal Chocolate Spectrum during the Time Interval not Containing the Main Pulse

Below we study the THz signal spectral properties in the time interval, which does not contain the main pulse, and continue to investigate the spectral resolution influence on its spectrum. This result is important for practice, because it shows the possibility of the substance absorption frequency detection in a highly noisy signal, in which the main pulse amplitude is comparable with, or less than a noise amplitude.

In [40] the sugar and chocolate spectral features presence was detected in the signal Chocolate using the ICC in the time interval *t* = [0, 25] ps, which contains the main pulse of the measured THz signal. Let us note that in [45] the possibility of the foreign inclusion detection in chocolate with the help of THz radiation was studied also for the main pulse.

Using sugar and chocolate as an example, in this Section we show that their spectral features detection is possible in the time interval *t* = [25, 110] ps, which does not contain the main pulse. For this purpose, we will use the standard transmitted THz signals Sucr10 and Choc10, which were measured under laboratory conditions at short distance (15 cm) in the Institute for Spectroscopy RAS, Troitsk, Russia (Choc10 signal) and in the Semiconductor Physics Institute, Vilnius, Lithuania (Sucr10 signal). The THz pulse shape and the corresponding signals’ Fourier spectra were shown in [37]. The absorption frequencies in these spectra are ν = 1.85 THz for Sucr10 and ν = 1.75 THz for Choc10.

**Figure 14:**
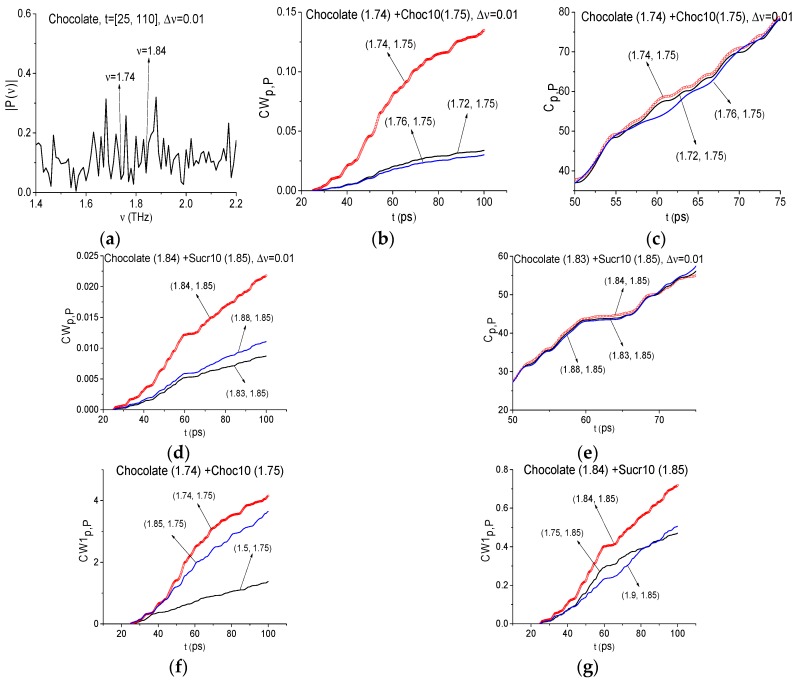
Chocolate signal spectrum (**a**) in the frequency range *ν* = [1.4, 2.2] THz calculated in the time interval *t* = [25, 110] ps with the spectral resolution Δ*ν* = 0.01 THz. The ICC’s *CW_p,P_* (**b**,**d**), *C_p,P_* (**c**,**e**), CW1_p*,P*_ (**f**,**g**) evolution, calculated for frequencies *ν* = 1.74 THz (**b**,**c**,**f**), 1.84 THz (**d**,**e**,**g**) for the signal Chocolate and Choc10 (**b**,**c**,**f**), Sucr10 (**d**,**e**,**g**) as the standard signals.

As an example, Figure 14a shows the corresponding signal spectrum in the frequency range ν = [1.4, 2.2] THz calculated with the spectral resolution Δ*ν* = 0.01 THz (the time interval was extended up to the duration *t* = 100 ps). As one can see, the spectrum contains minima at the frequencies ν = 1.84 THz and 1.74 THz, close to the absorption frequencies ν = 1.85 THz and ν = 1.75 THz of the standard sucrose and chocolate, correspondingly. Using the ICC’s *CW_p,P_*, *C_p,P_* (Figure 14b,c), one can detect the chocolate absorption frequency ν = 1.74 THz in the signal under investigation.

We see in Figure 14b,c that the line corresponding to the ICC *C_p,P_* calculated for the chocolate absorption frequency ν = 1.74 THz (c) lies above other lines in the time interval *t* = [50, 75] ps. Therefore, in this time interval the excitation of the corresponding energy transition takes place. Also by using the standard signal Sucr10, the frequency ν = 1.84 THz is detected as the sugar absorption frequency in the signal Chocolate, see Figure 14d,e. The criterion *L_p,P_* evolution in Figure 14 is not shown because the corresponding lines are almost coincide with each other for the considered FDRs *ν* = [1.72, 1.76] THz (b), (c) and *ν* = [1.83, 1.88] THz (d), (e). The evolution of ICC CW1_p*,P*_ (f), (g) confirms this result.

From the above there follows an important conclusion. If the standard substance is present in the sample, then at least two integral criteria (including the ICC *C_p,P_*) detect the standard substance absorption frequency in the signal under investigation. In Section 4.2 we demonstrated that the detection by only one criterion may be not enough for definite identification.

**Figure 15:**
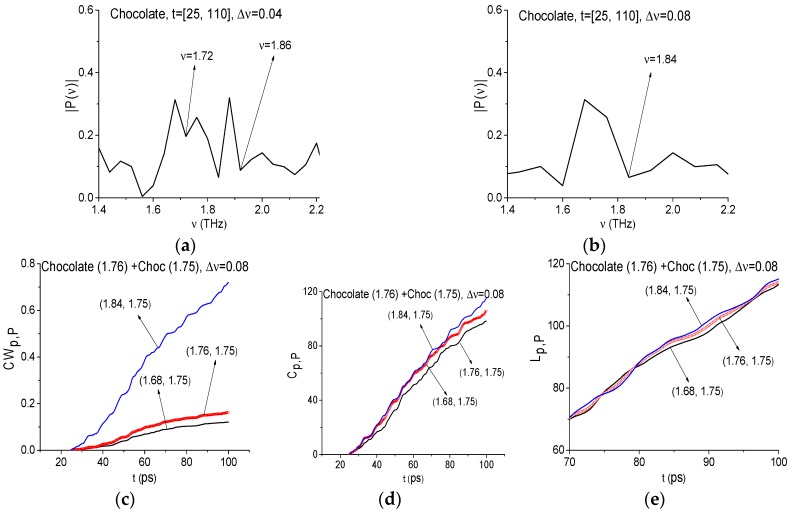
Signal Chocolate spectrum in the frequency range *ν* = [1.4, 2.2] THz calculated in the time interval *t* = [25, 110] ps with the spectral resolution Δ*ν* = 0.04 THz (**a**); 0.08 THz (**b**). The ICC’s *CW_p,P_* (**c**), *C_p,P_* (**d**), *L_p,P_* (**e**), calculated for frequency *ν* = 1.76 THz for the signal Chocolate and Choc10 as the standard one.

It should be noted that further spectral resolution decreasing up to Δ*ν* = 0.08 THz leads to the disappearance of the minimum close to the chocolate absorption frequency *ν* = 1.75 THz. The minimum at the frequency, close to sucrose absorption frequency *ν* = 1.85 THz, is present in the Chocolate spectrum in both cases (see Figure 15). The ICC’s *CW_p,P_*, *C_p,P_*, *L_p,P_* detect the presence of the sucrose absorption frequency in the signal Chocolate (not shown). The ICC’s *CW_p,P_* (c), *C_p,P_* (d), *L_p,P_* (f) do not detect the frequency *ν* = 1.75 THz as the chocolate absorption frequency in the Chocolate signal if one uses the with spectral resolution Δ*ν* = 0.08 THz. Therefore, decreasing the spectral resolution up to a certain value (in our case it is Δ*ν* = 0.08 THz) leads to the disappearance not only false frequencies but also of a part of the substance absorption frequencies with a small bandwidth.

**Figure 16:**
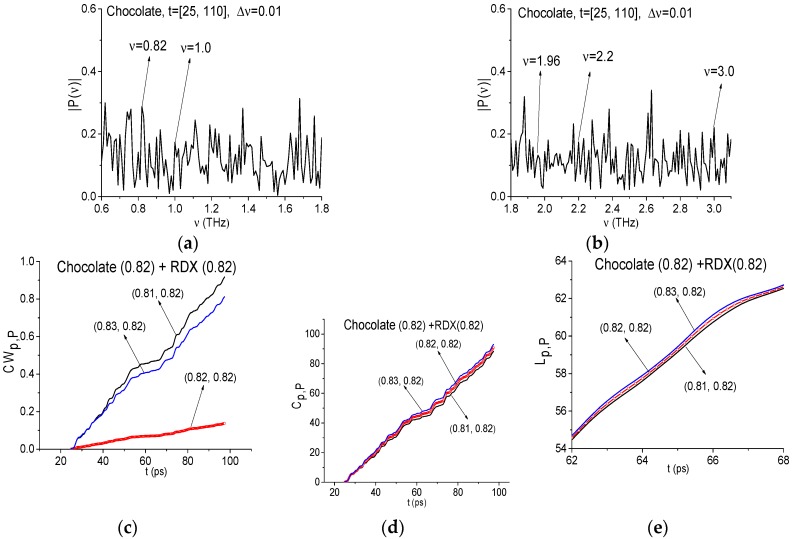
Signal Chocolate spectrum in the frequency ranges *ν* = [0.6, 1.8] (**a**); [1.8, 3.2] (**b**) calculated with the spectral resolution Δ*ν* = 0.01 THz. ICC *CW_p,P_* (**c**), *C_p,P_* (**d**), *L_p,P_* (**e**) for frequency *ν* = 0.82 THz and signal Chocolate and RDX_Air as a standard one.

Now we will show that the RDX spectral properties are absent in the signal Chocolate if we will analyze the signal in the time interval *t* = [25, 110] ps. In Figure 16a,b the signal Chocolate spectrum is presented for frequency ranges ν = [0.6, 1.8] THz (a) [1.8, 3.2] THz (b), calculated with the spectral resolution Δ*ν* = 0.01 THz (as above, the signal was extended up to the duration *t* = 100 ps and filled by zeroes). We see that the Chocolate signal spectrum minima at the frequencies ν = 0.82, 1.0, 1.95, 2.2 and 3.0 THz are absent. Therefore, the absorption of the signal at these frequencies is also absent. However, this can be caused by small-scale modulation due to noise. Therefore, it is necessary to analyze also the ICC.

The ICC’s at these frequencies also show the RDX absence in chocolate. Figure 16d–f shows the corresponding ICC’s evolution for frequency ν = 0.82 THz. Because of the very close location of the lines corresponding to the integral criterion *L_p,P_* (f), they are shown in the decreased time interval *t* = [62, 68] ps. All three criteria do not detect the frequency ν = 0.82 THz as RDX absorption frequency in the Chocolate signal. Similar results are also valid for other frequencies.

**Figure 17:**
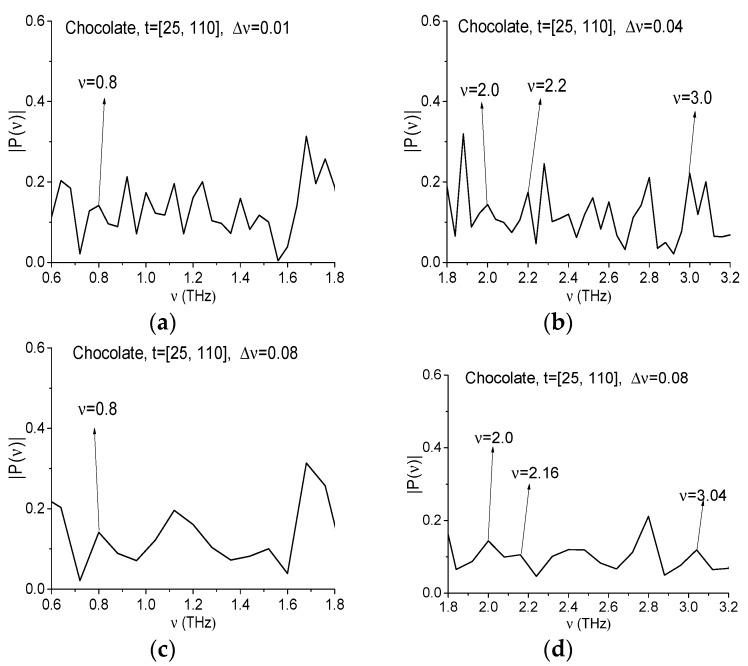
Signal Chocolate spectrum in the frequency ranges *ν* = [0.6, 1.8] (**a**); [1.8, 3.2] (**b**) calculated with the spectral resolution Δ*ν* = 0.04 THz (**a**,**b**), 0.08 THz (**c**,**d**).

In Figure 17a,c the spectrum of the l Chocolate signal is presented in the same frequency ranges ν = [0.6, 1.8] THz (a), (c), [1.8, 3.2] THz (b), (d) with the spectral resolution Δ ν = 0.04 THz (a), (b), 0.08 THz (c), (d).

In the figures (a)–(d) it can be seen that at frequencies ν = 0.8, 2.0, 2.2 (2.16 in (d)) and 3.0 (3.04 in (d)) THz in the Chocolate spectrum minima are absent, therefore the absorption at these frequencies is also absent. The ICC’s at these frequencies also confirm the RDX spectral features absence in the signal Chocolate (not shown). With decreasing of spectral resolution, the corresponding FRDs became larger. Thus, FDR for the frequency ν = 0.8 is equal to ν = [0.72, 0.88] THz (Δ*ν* = 0.04 THz), and ν = [0.72, 0.96] THz (Δ ν = 0.08 THz).

### 4.4. Spectral Resolution Influence on the Signal Chocolate Spectrum in the Partial Time Intervals Following the Main Pulse

It should be emphasized that for the substance detection it can also be effective to divide the interval *t* = [25, 110] ps into several parts with their subsequent analysis. Fourier spectra calculated in such intervals can contain minima corresponding to the absorption frequencies of hazardous substances. This follows from the fact that in the sample under investigation, the absorption at these frequencies may take place also after the main pulse ending. This phenomenon is due to relaxation occurring with different times of the molecules energy levels, which was excited by the broadband THz pulse because of the cascade mechanism. Since the spectral intensity of the emission frequencies and therefore, the corresponding absorption at these frequencies also is significantly lower than the corresponding spectral intensity for the main pulse, then at analyzing the spectrum during the total time interval, they could be easily missed because of the high spectral intensity of other frequencies. In the partial time interval, which includes these processes, the emission frequencies can be seen clearly.

**Figure 18:**
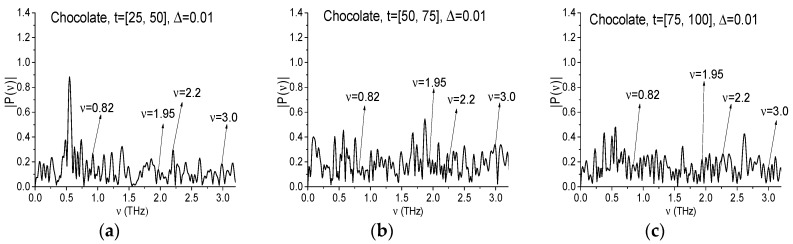

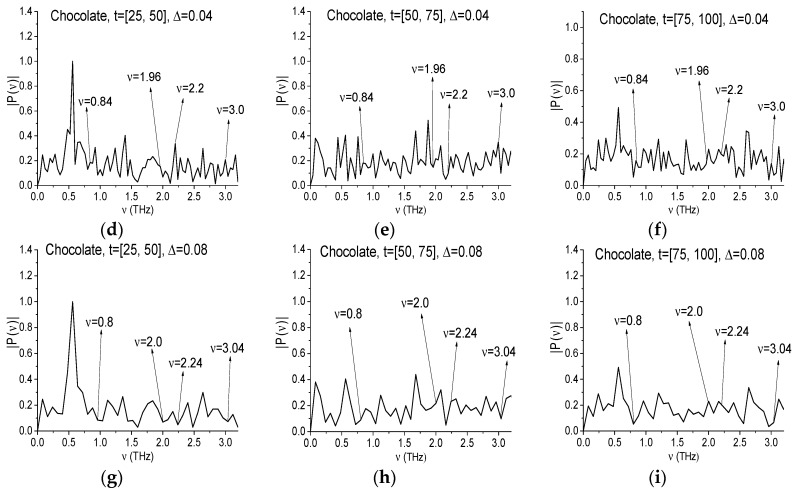
Signal Chocolate spectrum calculated in the time intervals *t* = [25, 50] (**a**,**d**), [50, 75] (**b**,**e**), [75, 100] (**c**,**f**) with the spectral resolution Δ*ν* = 0.01 THz (**a**–**c**), 0.04 THz (**d**–**f**), 0.08 THz (**g**–**i**).

As an illustration, Figure 18 demonstrates the Chocolate signal spectrum, calculated in the time intervals *t* = [25, 50] ps (a), [50, 75] ps (b), [75, 100] ps (c) with the duration *t* = 25 ps and with a spectral resolution Δ*ν* = 0.01 THz. One can see that at the frequencies ν = 0.82, 1.95 and 3.0 THz in the Chocolate signal spectrum there are the maxima in all three time intervals, as well as for the frequency ν = 2.2 THz in the time interval *t* = [25, 50] (a). However, in the time intervals *t* = [50, 75] ps (b), [75, 100] ps (c) the spectral intensity minimum at this frequency takes place, as well as at the frequency ν = 1.96 THz in the interval *t* = [50, 75] (b), which can show the RDX presence.

The spectral resolution decreasing up to Δ*ν* = 0.04 THz (Figure 18d–f) does not change the existence of spectral intensity maxima at the frequencies ν = 0.84, 3.0 THz, and the spectral maximum at the frequency ν = 2.2 THz in the first interval *t* = [25, 50] ps (d). At the frequencies ν = 1.96, 2.2 THz in the second time interval *t* = [50, 75] ps (e) the minima remain, but in the third interval *t* = [75, 100] ps (f) they disappear. In Figure 18g–i the corresponding spectra are calculated with the spectral resolution equal to Δ*ν* = 0.08 THz. We see again minima in Figure 18g at the frequencies ν = 0.8, 2.0, 2.24, 3.04 THz, which are close to the RDX absorption frequencies ν = 0.82, 1.95, 2.19, 3.0 THz. In (h) there is a minimum at the frequency ν = 3.04 THz, in (i)—at frequencies ν = 0.8, 3.04 THz. 

In order to understand the reason for the minimum’s appearance in the Chocolate signal spectra (Figure 18g–i), below we analyze the Reference spectrum in one of these partial time intervals at decreasing the spectral resolution.

**Figure 19:**
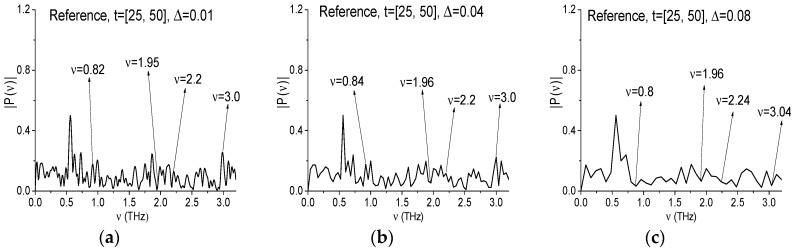
Reference spectrum calculated in the time interval *t* = [25, 50] with spectral resolution Δ*ν* = 0.01 THz (**a**), 0.04 THz (**b**), 0.08 THz (**c**).

Figure 19 shows the Reference spectrum calculated in the interval *t* = [25, 50] ps with the spectral resolution equal to Δ*ν* = 0.01 THz (a), 0.04 THz (a) and 0.08 THz (c). In (a) (the spectral resolution Δ*ν* = 0.01 THz) the minima at the frequencies ν = 0.82, 1.95, 2.2, 3.0 THz are absent. The Reference spectrum in (b) (the spectral resolution Δ*ν* = 0.04 THz) also shows the absence of minima at the frequencies ν = 0.84, 2.2, 3.0 THz. Thus, the environmental influence on these minima appearance in the Chocolate signal spectrum (Figure 18a,d) in the time interval *t* = [25, 50] ps is absent.

However, in Figure 19c (the spectral resolution is decreased up to Δ*ν* = 0.08 THz) the Reference spectrum minima (or values closest to minima) appear again at the frequencies close to RDX absorption frequencies ν = 0.8, 1.96, 2.24, 3.0 THz. The same Reference spectrum minima can be seen for the corresponding Chocolate signal spectrum in Figure 18g.

The reason for this conclusion is a consequence of strong spectral resolution decreasing, which leads to big errors in the spectrum. In turn, such errors arise because the half-width of the corresponding absorption lines of the Chocolate signal at the frequencies ν = 0.82, 1.95, 2.2, 3.0 THz are less than 0.04 THz, which does not correspond to the RDX spectrum (see Figure 10a–c). Let us remind ourselves that for the signal RDX_Air, the half-width of the absorption line at the frequency ν = 0.82 THz is not less than 0.1 THz [3,4,5]. Therefore, if RDX is present in the chocolate, then we have to observe the minimum at the frequencies close to ν = 0.82 THz (ν = 0.8 or 0.84 THz) in all spectra (a)–(f). *L_p,P_*.

**Figure 20:**
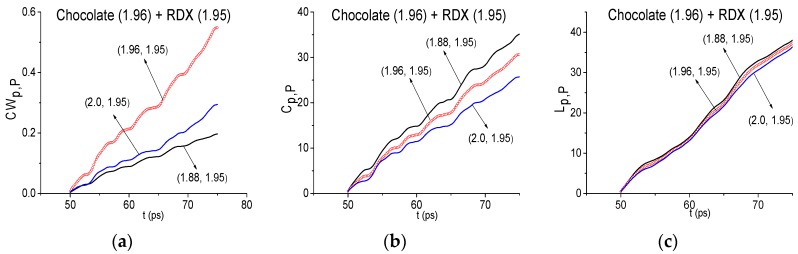
ICC’s *CW_p,P_* (**a**), *C_p,P_* (**b**), *L_p,P_* (**c**) calculated for frequency *ν* = 1.96 THz with the spectral resolution Δ*ν* = 0.04 THz for signal Chocolate and RDX_Air as a standard one.

Nevertheless, the spectral resolution decreasing in the Chocolate signal spectrum calculation in the partial time intervals cannot eliminate all the false absorption frequencies therein. So, it is necessary to use the ICC’s for their elimination. With this aim Figure 20a–c show the evolution of the ICC’s *CW_p,P_* (a), *C_p,P_* (b), *L_p,P_* (c) in the time interval *t* = [50, 75] ps for the frequency ν = 1.96 THz with the spectral resolution Δ*ν* = 0.04 THz. The values of ICC *CW_p,P_* calculated for the frequency ν = 1.96 THz (a), lie above the others. But in the corresponding Reference spectrum there is a minimum at this frequency (not shown), which is close to water vapor absorption frequency ν = 1.92 [16] (the corresponding spectral resolution in [16] was equal to Δ*ν* = 0.01 THz). Therefore, this result can be explained by the influence of water vapor containing in the air. At the same time, the ICC’s *C_p,P_* and *L_p,P_* do not detect the frequency ν = 1.96 THz as the RDX absorption frequency in the signal Chocolate. Similar results occur for the frequency ν = 2.2 THz.

Thus, the spectral properties analysis for a signal under investigation in the time interval that does not contain the main pulse, also provides important information. Using the spectrum analysis and ICC’s *C_p,P_*, *L_p,P_* the spectral features presence of sugar and chocolate, as well as the absence of dangerous substances in the Chocolate signal are shown with the spectral resolution Δ*ν* = 0.01, 0.04 THz. At the same time the spectral resolution decreasing up to Δ*ν* = 0.08 THz in some cases leads to incorrect results.

## 5. Algorithm of Spectral Resolution Using at the Substance Detection

Summing up our study of the noisy THz signals spectra in various time intervals, we propose the following procedure for the analysis of such spectra for substance detection and identification. 

At the first step, we calculate the THz main pulse spectrum with low spectral resolution, which is 80%–100% of the corresponding half-width of the substance standard absorption line. For example, for the substance RDX detection in the Chocolate signal, this value was equal to Δ*ν* = 0.08 THz (or 0.1 THz) for the RDX absorption frequency ν = 0.82 THz. The spectral minimum absence at the substance absorption frequency means its absence in the spectrum calculated with increased spectral resolution. Thus, in the example with the substance RDX, it is not necessary to check up the presence of this RDX absorption line in the Chocolate signal spectrum calculated with higher spectral resolution (for example, Δ*ν* = 0.04 THz). The presence of other substance absorption frequencies in the investigated spectrum is verified in a similar way.

If the spectral minimum at the standard absorption frequency is present in the spectrum under investigation and calculated with spectral resolution about of 40–80% of the half-width of the corresponding absorption line from database, then we analyze the Reference spectrum calculated with the same spectral resolution and in the same time interval. The Reference spectrum minimum presence at the same frequency means that this minimum in the spectrum of the signal under investigation is caused by the environment influence or water vapor absorption. In the case if the Reference spectrum minimum is absent, then we use the several ICC simultaneously for the detailed analysis. To confirm the standard substance presence in the sample, at least two integral correlation criteria (including the ICC *C_p,P_* and/or *L_p,P_*) have to detect the standard substance absorption frequency in the signal under investigation. In the case of low detection contrast, it can be increased in some cases at decreasing the spectral resolution.

The check-up procedure for the minima close to the standard substance absorption frequencies in the time intervals, which do not contain the main pulse of the investigated signal, is similar to the procedure described above. This preliminary testing allows us to speed up the detection and identification process.

The optimal option at the analysis of the spectral properties of the noisy THz signals measured under real conditions with the help of the SDA-ICC method, seems to be a spectral resolution equal to 40% of the half-width of the corresponding substance standard absorption line. It is recommended for the detection and identification of substances in the time intervals containing and not containing the main pulse. All recommendations are valid for the emission frequency investigation as well.

## 6. Conclusions

We have demonstrated the essential limitations of the standard THz-TDS method for substance analysis based on a comparison of the substance spectrum under investigation and the standard substance spectrum from a database. Its use under real conditions (at long distances more than 3 m and at high relative humidity) leads to hazardous substances false detection in the neutral samples.

It is shown that increasing spectral resolution for the high-noise signal leads to the small-scale modulation observing in the signal and its spectrum, which are caused by packing material, or the structure of material. It is important in practice that decreasing spectral resolution allows us in some cases to exclude from consideration the false absorption frequencies, as well as to detect the frequencies caused by water vapor absorption. At the same time, we showed that spectral resolution that is decreasing too strongly (up to Δ*ν* = 0.08 THz, for example, in the case of the substance RDX detection in the Chocolate signal) leads to large errors in the spectrum; one can see many false absorption frequencies in the spectrum. Therefore, the optimal spectral resolution depends on a bandwidth of the absorption line for database substance under consideration. For example, taking into account the half-width of the spectral line for the RDX absorption frequency ν = 0.82 THz (its value is not less than 0.1 THz [3,4,5], see Section 4.2), the optimal spectral resolution is the value of Δ*ν* = 0.04 THz, or 40% of the corresponding spectral line half-width.

The SDA-method together with the ICC, which takes into account the spectral intensities changes in time, is free from these disadvantages and allows us to detect the presence of the standard substance spectral features presence in the signal under investigation. In order to increase the detection reliability, it is advisable first to decrease the spectral resolution up to the values not more than 40% of the corresponding spectral line half-width and then to use several integral criteria simultaneously. The ICC detection contrast does not degrade with decreasing spectral resolution, and in some cases detection contrast can be increased.

Substance detection can be carried out using the main THz pulse, and a signal part behind it. This opens up the substance identification possibility at distances of about 10 m, since in this case the noisy signal under analysis will not contain a pronounced THz pulse. Therefore, the proposed algorithm can be applied for remote sensing of materials.

## References

[1] Federici J.F., Schulkin B., Huang F., Gary D., Barat R., Oliveira F., Zimdars D. (2005). THz imaging and sensing for security applications—Explosives, weapons and drugs. Semicond. Sci. Technol..

[2] Lu M., Shen J., Li N., Zhang Y., Zhang C., Liang L., Xu X. (2006). Detection and identification of illicit drugs using terahertz imaging. J. Appl. Phys..

[3] Shen Y.C., Lo T., Taday P.E., Cole B.E., Tribe W.R., Kemp M.C. (2005). Detection and identification of explosives using terahertz pulsed spectroscopic imaging. Appl. Phys. Lett..

[4] Leahy-Hoppa M.R., Fitch M.J., Zheng X., Hayden L.M., Osiander R. (2007). Wideband terahertz spectroscopy of explosives. Chem. Phys. Lett..

[5] Chen J., Chen Y., Zhao H., Bastiaans G.J., Zhang X.-C. (2007). Absorption coefficients of selected explosives and related compounds in the range of 0.1–2.8 THz. Opt. Express.

[6] Davies A.G., Burnett A.D., Fan W., Linfield E.H., Cunningham J.E. (2008). Terahertz spectroscopy of explosives and drugs. Mater. Today.

[7] Choi K., Hong T., Sim K.I., Ha T., Park B.C., Chung J.H., Cho S.G., Kim J.H. (2014). Reflection terahertz time-domain spectroscopy of RDX and HMX explosives. Appl. Phys..

[8] Ergün S., Sönmez S. (2015). Terahertz technology for military applications. J. Mil. Inf. Sci..

[9] Katz G., Zybin S., Goddard W.A., Zeiri Y., Kosloff R. (2014). Direct MD simulations of terahertz absorption and 2D spectroscopy applied to explosive crystals. J. Phys. Chem. Lett..

[10] Bou-Sleiman J., Perraud J.B., Bousquet B., Guillet J.P., Palka N., Mounaix P. (2015). Discrimination and identification of RDX/PETN explosives by chemometrics applied to terahertz time-domain spectral imaging. Proc. SPIE.

[11] Choi J., Ryu S.Y., Kwon W.S., Kim K.S., Kim S. (2013). Compound explosives detection and component analysis via terahertz time-domain spectroscopy. J. Opt. Soc. Korea.

[12] Xiong W., Shen J. (2010). Fingerprint extraction from interference destruction terahertz spectrum. Opt. Express.

[13] Ortolani M., Lee J.S., Schade U., Hübers H.-W. (2008). Surface roughness effects on the terahertz reflectance of pure explosive materials. Appl. Phys. Lett..

[14] Slocum D.M., Slingerland E.J., Giles R.H., Goyette T.M. (2013). Atmospheric absorption of terahertz radiation and water vapor continuum effects. J. Quant. Spectrosc. Radiat. Transfer.

[15] Kemp M.C. (2011). Explosives detection by terahertz spectroscopy—A bridge too far?. IEEE Trans. Terahertz Sci. Technol..

[16] Van Rheenen A.D., Haakestad M.W. (2011). Detection and identification of explosives hidden under barrier materials—What are the THz-technology challenges?. Proc. SPIE.

[17] Puc U., Abina A., Rutar M., Zidanšek A., Jeglič A., Valušis G. (2015). Terahertz spectroscopic identification of explosive and drug simulants concealed by various hiding techniques. Appl. Opt..

[18] Duling I., Zimdars D. (2009). Terahertz imaging: Revealing hidden defects. Nat. Photonics.

[19] Kawase K., Shibuya T., Hayashi S.I., Suizu K. (2010). THz imaging techniques for nondestructive inspections. C. R. Phys..

[20] Zimdars D., White J.S., Stuk G., Chernovski A., Fichter G., Williamson S. (2006). Large area terahertz imaging and non-destructive evaluation applications. Insight.

[21] Pickwell E., Wallace V.P. (2006). Biomedical applications of terahertz technology. J. Phys. D.

[22] Schirmer M., Fujio M., Minami M., Miura J., Araki T., Yasui T. (2010). Biomedical applications of a real-time terahertz color scanner. Biomed. Opt. Express.

[23] Ouchi T., Kajiki K., Koizumi T., Itsuji T., Koyama Y., Sekiguchi R., Kawase K. (2014). Terahertz imaging system for medical applications and related high efficiency terahertz devices. J. Infrared Millim. Terahertz Waves.

[24] Yang X., Zhao X., Yang K., Liu Y., Liu Y., Fu W., Luo Y. (2016). Biomedical applications of terahertz spectroscopy and imaging. Trends Biotechnol..

[25] Balčytis A., Ryu M., Wang X., Novelli F., Seniutinas G., Du S., Wang X., Li J., Davis J., Appadoo D., Morikawa J. (2017). Silk: Optical properties over 12.6 octaves THz-IR-visible-UV range. Materials.

[26] Shen Y.C. (2011). Terahertz pulsed spectroscopy and imaging for pharmaceutical applications: A review. Int. J. Pharm..

[27] Ok G., Park K., Kim H.J., Chun H.S., Choi S.W. (2014). High-speed terahertz imaging toward food quality inspection. Appl. Opt..

[28] Shen Y.C., Taday P.F., Newnham D.A., Pepper M. (2005). Chemical mapping using reflection terahertz pulsed imaging. Semicond. Sci. Technol..

[29] McIntosh A.I., Yang B., Goldup S.M., Watkinson M., Donnan R.S. (2012). Terahertz spectroscopy: A powerful new tool for the chemical sciences?. Chem. Soc. Rev..

[30] El Hadda J., Bousquet B., Canioni L., Mounaix P. (2013). Review in terahertz spectral analysis. TrAC Trends Anal. Chem..

[31] Ahi K., Anwar M. (2016). Advanced terahertz techniques for quality control and counterfeit detection. Proc. SPIE.

[32] Ahi K., Anwar M. (2016). Developing terahertz imaging equation and enhancement of the resolution of terahertz images using deconvolution. Proc. SPIE.

[33] Ahi K. (2017). Review of GaN-based devices for terahertz operation. Opt. Eng..

[34] Trofimov V.A., Varentsova S.A., Zhang C., Shen J., Zhou Q., Shi Y. (2011). 2D signature for identification of drugs. Proc. SPIE.

[35] Trofimov V.A., Varentsova S.A., Chen J., Zhang X.-C. (2009). Identification of explosive media using their spectrum dynamics under the action of THz pulse. Proc. SPIE.

[36] Trofimov V.A., Varentsova S.A., Szustakowski M., Palka N. (2013). Influence of surface of explosive on its detection and identification using the SDA method for analysis of the reflected THz signal. Proc. SPIE.

[37] Trofimov V.A., Varentsova S.A., Trofimov V.V., Tikhomirov V.V. (2014). Peculiarities of the detection and identification of substance at long distance. Proc. SPIE.

[38] Trofimov V.A., Varentsova S.A. (2015). An effective method for substance detection using the broad spectrum THz signal: A “Terahertz nose”. Sensors.

[39] Trofimov V.A., Varentsova S.A. (2016). Essential limitations of the standard THz TDS method for substance detection and identification and a way of overcoming them. Sensors.

[40] Trofimov V.A., Varentsova S.A. (2016). False detection of dangerous and neutral substances in commonly used materials by means of the standard THz time domain spectroscopy. J. Eur. Opt. Soc..

[41] Trofimov V.A., Varentsova S.A. (2016). Detection and identification of drugs under real conditions by using noisy terahertz broadband pulse. Appl. Opt..

[42] Trofimov V.A., Varentsova S.A., Zakharova I.G., Zagursky D.Y. (2016). How can we distinguish between simulants and hazardous substances under real conditions?. Proc. SPIE.

[43] Sun J.-H., Shen J.-L., Liang L.-S., Xu X.-Y., Liu H.-B., Zhang C.-L. (2005). Experimental investigation on terahertz spectra of amphetamine type stimulants. Chin. Phys. Lett..

[44] Trofimov V.A., Varentsova S.A., Trofimov V.V. (2014). Possibility of the detection and identification of substance at long distance at using broad THz pulse. Proc. SPIE.

[45] Jördens C., Koch M. (2008). Detection of foreign bodies in chocolate with pulsed terahertz spectroscopy. Opt. Eng..

